# Analysis of the Differentially Expressed Genes Induced by Cisplatin Resistance in Oral Squamous Cell Carcinomas and Their Interaction

**DOI:** 10.3389/fgene.2019.01328

**Published:** 2020-01-23

**Authors:** Hua-Tao Wu, Wen-Tian Chen, Guan-Wu Li, Jia-Xin Shen, Qian-Qian Ye, Man-Li Zhang, Wen-Jia Chen, Jing Liu

**Affiliations:** ^1^Department of General Surgery, The First Affiliated Hospital of Shantou University Medical College, Shantou, China; ^2^Department of Physiology, Shantou University Medical College, Shantou, China; ^3^Open Laboratory for Tumor Molecular Biology, Department of Biochemistry, The Key Lab of Molecular Biology for High Cancer Incidence Coastal Chaoshan Area, Shantou University Medical College, Shantou, China; ^4^Department of Hematology, The First Affiliated Hospital of Shantou University Medical College, Shantou, China; ^5^Chang Jiang Scholar’s Laboratory/Guangdong Provincial Key Laboratory for Diagnosis and Treatment of Breast Cancer, Shantou University Medical College, Shantou, China

**Keywords:** differentially expressed genes, resistance, oral squamous cell carcinomas, cisplatin, miRNA

## Abstract

**Background:**

Oral squamous cell carcinoma (OSCC) is a solid tumor, which originates from squamous epithelium, with about 400,000 new-cases/year worldwidely. Presently, chemoradiotherapy is the most important adjuvant treatment for OSCC, mostly in advanced tumors. However, clinical resistance to chemotherapy still leads to poor prognosis of OSCC patients. Via high-throughput analysis of gene expression database of OSCC, we investigated the molecular mechanisms underlying cisplatin resistance in OSCC, analyzing the differentially expressed genes (DEGs) and their regulatory relationship, to clarify the molecular basis of OSCC chemotherapy resistance and provide a theoretical foundation for the treatment of patients with OSCC and individualized therapeutic targets accurately.

**Methods:**

Datasets related to “OSCC” and “cisplatin resistance” (GSE111585 and GSE115119) were downloaded from the GEO database and analyzed by GEO2R. Venn diagram was used to obtain drug-resistance-related DEGs. Functional enrichment analysis and Kyoto encyclopedia of genes and genomes (KEGG) pathway analysis were performed on DEGs using The Database for Annotation, Visualization and Integrated Discovery (DAVID) software. Protein–protein interaction (PPI) network was constructed by STRING (search tool for recurring instances of neighbouring genes) database. Potential target genes of miRNA were predicted *via* miRDB, and cBioportal was used to analyze the function and survival of the potential functional genes.

**Results:**

Forty-eight upregulated DEGs and 49 downregulated DEGs were obtained from the datasets, with cutoff as *p <* 0.01 and |log FC| > 1. The DEGs in OSCC mainly enriched in cell proliferation regulation, and chemokine activity. In PPI network with hub score > 300, the hub genes were identified as *NOTCH1*, *JUN*, *CTNNB1*, *CEBPA*, and *ETS1*. Among miRNA–mRNA targeting regulatory network, hsa-mir-200c-3p, hsa-mir-200b-3p, hsa-mir-429, and hsa-mir-139-5p were found to simultaneously regulate multiple hub genes. Survival analysis showed that patients with high *CTNNB*1 or low *CEBPA* expression had poor outcome.

**Conclusions:**

In the OSCC cisplatin-resistant cell lines, *NOTCH1*, *JUN*, *CTNNB1*, *CEBPA*, and *ETS1* were found as the hub genes involved in regulating the cisplatin resistance of OSCC. Members of the miR-200 family may reverse drug resistance of OSCC cells by regulating the hub genes, which can act as potential targets for the treatment of OSCC patients with cisplatin resistance.

## Introduction

Head and neck squamous cell carcinoma (HNSCC), the sixth most common malignant tumor in the world (Kim et al., 2011a), is an important public health issue worldwide. Among the total HNSCC cases, 30% are oral squamous cell carcinoma (OSCC) cases (World Health, 2003; Petersen, 2003a; Petersen, 2003b). In 2012, about 145,000 patients with OSCC died worldwide, with a mortality rate of 1.8% (Petersen, 2005; Kim et al., 2011a; Ong et al., 2016). Interestingly, OSCC is one of the three most common malignancies in Central and South Asia. In India, the age-standardized incidence of OSCC is 12.6 per 100,000 people (Petersen, 2005). According to statistics, the incidence of OSCC has increased sharply in several countries and regions, including Denmark, France, Germany, Scotland, and Central and Eastern Europe (Petersen, 2005).

OSCC can occur in different areas of the mouth and tongue, including lips, alveolar ridge, oral floor, oral tongue, hard palate, posterior molars triangle, and buccal mucosa, lined by squamous epithelium and scattered in smaller salivary glands and lymphatic drainage pathways. OSCC is common in the elder people with a history of tobacco and alcohol usage, with malignant tumors or somatic cell mutation by inducing DNA damage (Leemans et al., 2011). Although surgery is the main treatment strategy for OSCC, chemoradiotherapy is also an effective method, especially for advanced tumors. However, drug resistance due to unraveled molecular mechanisms significantly reduces the survival of OSCC patients.

Since the first miRNA— lin-4 was identified in 1993, miRNAs have attracted the attention of researchers in the field of gene expression regulation and gene therapy (Liang et al., 2014). By inhibition of RNA translation or degradation of target mRNA, miRNAs act as negative gene regulators at the post-transcriptional level (Sakai et al., 2013). Importantly, miRNAs can simultaneously modulate many target genes, such as tumor suppressors or oncogenes, widely influencing the phenotype of malignant tumors. Since miRNAs have been found to have important role in various aspects of malignant tumors, including oncogenesis, proliferation, metastasis, multidrug resistance, self-renewal, and differentiation of malignant stem cells (Wu et al., 2014), they may represent a new set of therapeutic target biomarkers for finding multidrug resistance in malignant tumors (Hong et al., 2013).

In this study, the potential molecular mechanisms of cisplatin resistance of OSCC were studied by using high-throughput gene expression database. The differentially expressed genes (DEGs) in OSCC and their regulatory relationships were analyzed, in order to elucidate the molecular basis of OSCC chemotherapy resistance, and to provide theoretical basis and individualized precise therapeutic targets for the treatment of OSCC patients.

## Materials and Methods

### Microarray Datasets

“OSCC” and “cisplatin resistance” were used as the keywords for searching the GEO database, and GSE111585 and GSE115119 were downloaded as the gene expression data sets for cisplatin resistance in OSCC; the platforms used were GPL14715 and GPL16955.

GSE111585 included six samples of SCC9 cells and was divided into normal group and drug resistance group (Lin et al., 2018). GSE115119 contained four samples of CAL-27 cells, with normal group and drug resistant group. Both SCC9 and CAL-27 are human OSCC cell lines.

### Data Analysis and Differential Expressed Gene Acquisition

Limma package of R software (GEO2R) was used for analysis of the original datasets. |log FC| > 1 and *p* value < 0.01 were defined as the cutoff values for further analysis of DEGs. Volcano maps were constructed by SangerBox software.

Furthermore, the list of oncogenes (http://ongene.bioinfo-minzhao.org/) (Liu et al., 2017) and tumor-suppressor genes (https://bioinfo.uth.edu/TSGene/index.html) (Zhao et al., 2016) provided potential functional roles of genes in cancer process. To obtain DEGs in cisplatin-resistant OSCC cells, Venn package (http://bioinformatics.psb.ugent.be/webtools/Venn/) was used to draw the intersection of the up-regulated or down-regulated genes in the datasets with oncogenes or tumor-suppressor genes, respectively.

### Functional Enrichment Analysis of DEGs

Gene Ontology (GO) provides a computational model of biological systems, from the molecular to the organism level, across different species in the following three categories: biological process (BP), molecular function (MF), and cellular component (CC) (Thomas, 2017). Kyoto encyclopedia of genes and genomes (KEGG) is a database for high-level functions and utilities of the biological systems, based on molecular-level information of genome sequencing and other high-throughput experimental technologies (Kanehisa et al., 2017). DAVID Bioinformatics Resources 6.8 (https://david.ncifcrf.gov/) comprises a comprehensive set of functional annotation tools for functional enrichment analysis of gene groups (Huang da et al., 2009). To identify the biological significance of DEGs in cisplatin-resistant OSCC cells, DAVID 6.8 was used to analyze GO function and KEGG pathway enrichment, with the enrichment standard as *p* < 0.05.

### Protein–Protein Interaction Network of DEGs

Protein–protein interaction (PPI) network analysis is helpful to investigate the molecular mechanisms of diseases and discover new drug targets from a systematic perspective. STRING 11.0 (https://string-db.org/), covering more than 5,000 organisms with known and predicted protein–protein interactions, provides direct (physical) and indirect (functional) association (Szklarczyk et al., 2019). The PPI analysis of DEGs was performed by STRING 11.0, and the results were analyzed by Cytoscape 3.7.1. Furthermore, the cytoHubba plug in was used to calculate the interaction coefficient score between the DEGs. The top genes with hub score > 300 were identified as the hub genes with high connectivity in the PPI network.

### Predicting Hub Gene-Related miRNAs

MicroRNAs (miRNAs), small non-coding RNA molecules with highly conserved regions, regulate the expression of target genes by binding to the 3’-untranslated regions (3’-UTR) of specific mRNAs, involved in many physiological and disease processes. Each miRNA is thought to regulate multiple genes with enormous potential regulatory circuitry afforded by miRNA (Lim et al., 2003). To identify the potential miRNA–mRNA interaction in the network of the hub genes, miRDB (http://mirdb.org/), an online resource for miRNA target prediction and functional annotation (Wong and Wang, 2015), was used to predict the hub gene-related miRNAs, and the miRNA–mRNA regulatory network was constructed by Cytoscape 3.7.1.

### Expression and Survival Analysis of Hub Genes

The Oncomine database (https://www.oncomine.org/resource/login.html), an online cancer microarray database-mining platform (Rhodes et al., 2004), was used to investigate the difference in transcriptional levels of the hub genes in HNSCC and normal tissues.

As mutations of oncogenes and/or tumor-suppressor genes are frequent in tumor tissues, the Human Protein Atlas (http://www.proteinatlas.org/) was analyzed for the prognostic values of the hub genes (Uhlen et al., 2017), and cBioportal database (http://www.cbioportal.org/), an open-access online resource for multi-dimension analysis of data from The Cancer Genome Atlas (TCGA) (Gao et al., 2013), was used to analyze the effects of mutations in hub genes on the survival of patients with OSCC (MD Anderson, Cancer Discov, 2013).

## Results

### Difference of Gene Expression Between Parental and Cisplatin-Resistant OSCC Cells

**Figure 1:**
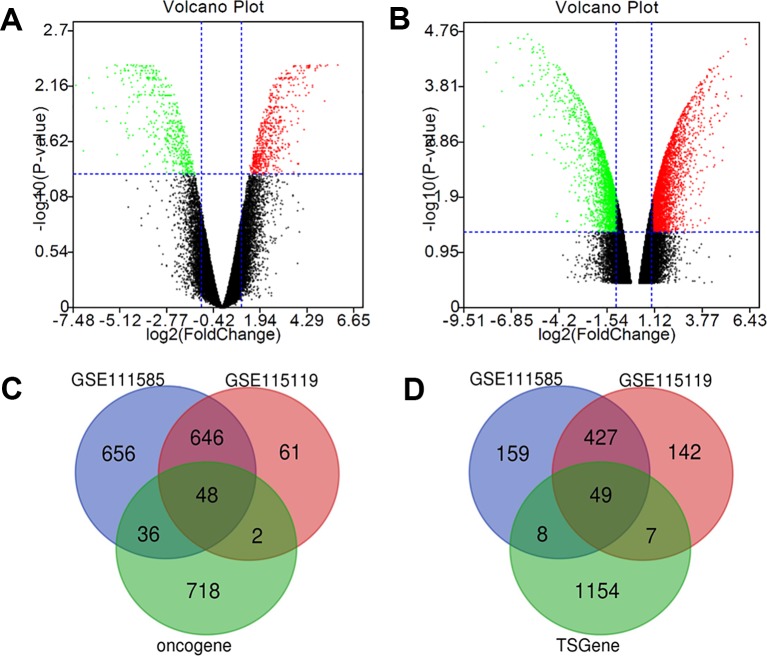
Identification of cisplatin-resistant DEGs in OSCC. **(A)** Volcano map of GSE111585. **(B)** Volcano map of GSE115119. **(C)** 48 up-regulated DEGs was selected based on the intersection between up-regulated gene in GSE111585/GSE115119 and oncogenes. **(D)** Forty-nine down-regulated DEGs was selected based on the intersection between down-regulated gene in GSE111585/GSE115119 and tumor-suppressor genes.

The gene expression microarray datasets, GSE111585 and GSE115119 were downloaded from GEO datasets with paired parental and cisplatin-resistant OSCC cells. As shown in **Figure 1**, the expression of most genes in cisplatin-resistant OSCC cells was similar to that of the parental OSCC cells. Cluster analysis by R software (|log FC| > 1 and *p* value < 0.01 as the cutoff) revealed 1,386 up-regulated genes and 643 down-regulated genes in cisplatin-resistant OSCC cells compared with parental OSCC cells in GSE111585 (**Figure 1A**), and 757 up-regulated genes and 625 down-regulated genes in cisplatin-resistant OSCC cells compared with parental OSCC cells in GSE115119 (**Figure 1B**).

The intersection between DEGs and the list of oncogenes drawn by Venn software showed 48 up-regulated DEGs (**Figure 1C**), and 49 down-regulated DEGs were obtained *via* intersection between down-regulated genes and the list of tumor-suppressor genes (**Figure 1D**).

### Close Association of the DEGs With the Regulation of Transcription and microRNAs in Cancers

**Figure 2:**
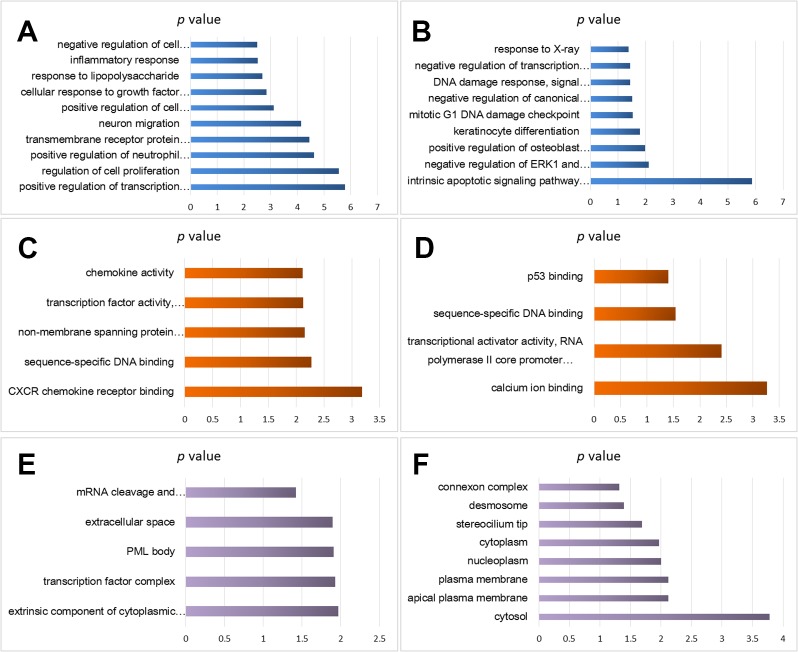
Functional enrichment analysis of cisplatin-resistant DEGs in OSCC. **(A)** BP analysis of up-regulated DEGs. **(B)** BP analysis of down-regulated DEGs. **(C)** MF analysis of up-regulated DEGs. **(D)** MF analysis of down-regulated DEGs. **(E)** CC analysis of up-regulated DEGs. **(F)** CC analysis of down-regulated DEGs.

Using the DAVID analysis software, functional enrichment analyses (BP, MF, and CC) of the DEGs were done. BP enrichment showed that the up-regulated DEGs were mainly enriched in cell proliferation regulation, inflammatory reaction, lipopolysaccharide, cells in response to growth factors to stimulate, neuronal migration, transmembrane receptor protein tyrosine kinase signaling pathway, and transcription of RNA polymerase II promoter (**Figure 2A**), whereas down-regulated DEGs were significantly enriched mainly in the following GO terms: response to X-ray, RNA polymerase II promoter negative transcription regulation, and DNA damage response (**Figure 2B**).

MF enrichment showed that the up-regulated DEGs were significantly enriched in chemokine activity, transcription factor activity, sequence specific DNA binding, non-membrane crossing protein tyrosine kinase activity, and sequence specific DNA binding (**Figure 2C**), and the down-regulated DEGs were enriched in p53 binding, sequence specific DNA binding, transcriptional activator activity, and RNA polymerase II hub promoter proximal region sequence specific binding (**Figure 2D**).

CC analysis predicted close association between the up-regulated DEGs and the following GO terms: mRNA cutting, polyadenylation specific factor complex, extracellular space, promyelocytic leukemia proteome, and transcription factor complex (**Figure 2E**), and significant relation was found between the down-regulated DEGs and the following GO terms: junction complex, desmosomes, ciliated tips, cytoplasm, nuclear cytoplasm, and plasma membrane (**Figure 2F**).

**Figure 3:**
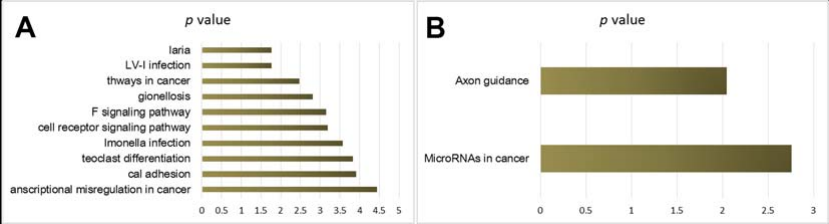
KEGG pathway analysis of cisplatin-resistant DEGs in OSCC. **(A)** KEGG of up-regulated DEGs. **(B)** KEGG of down-regulated DEGs.

KEGG pathway analysis provided the potential function cluster of DEGs, showing that the up-regulated DEGs were clustered in malaria, human T-cell leukemia virus type I, the way of malignant tumor, legionella infection disease, TNF signaling pathways, and T-cell receptors signaling pathways (**Figure 3A**), whereas the down-regulated DEGs were significantly concentrated in axon guidance and microRNAs in cancers (**Figure 3B**).

### Identification of Hub Genes Through PPI Network of DEGs

**Figure 4:**
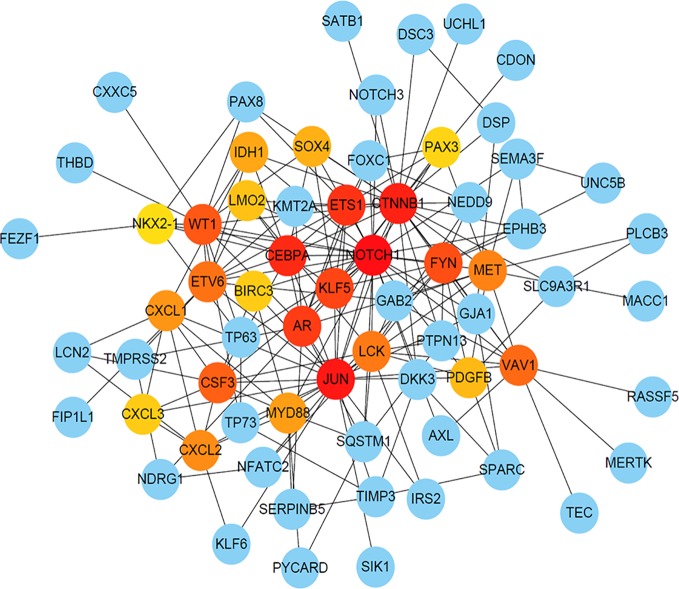
The PPI network of DEGs with Hub score. The dark color indicates high hub score, and the light color predicts low hub score.

To further analyze the correlation between DEGs in cisplatin-resistant OSCC cells, STRING was used to construct PPI network showing close relationship between the DEGs (**Supplemental Figure 1**), and their hub score was calculated. The genes with high hub score were predicted to have a strong association with other genes (shown in dark color in the figures). As shown in **Figure 4**, based on the cutoff hub score > 300, the following five genes were selected as the hub genes: *NOTCH1*, *JUN*, *CTNNB1*, *CEBPA*, and *ETS1*.

### Construction of miRNA–mRNA Network Based on Predicting miRNA-Target Genes

**Figure 5:**
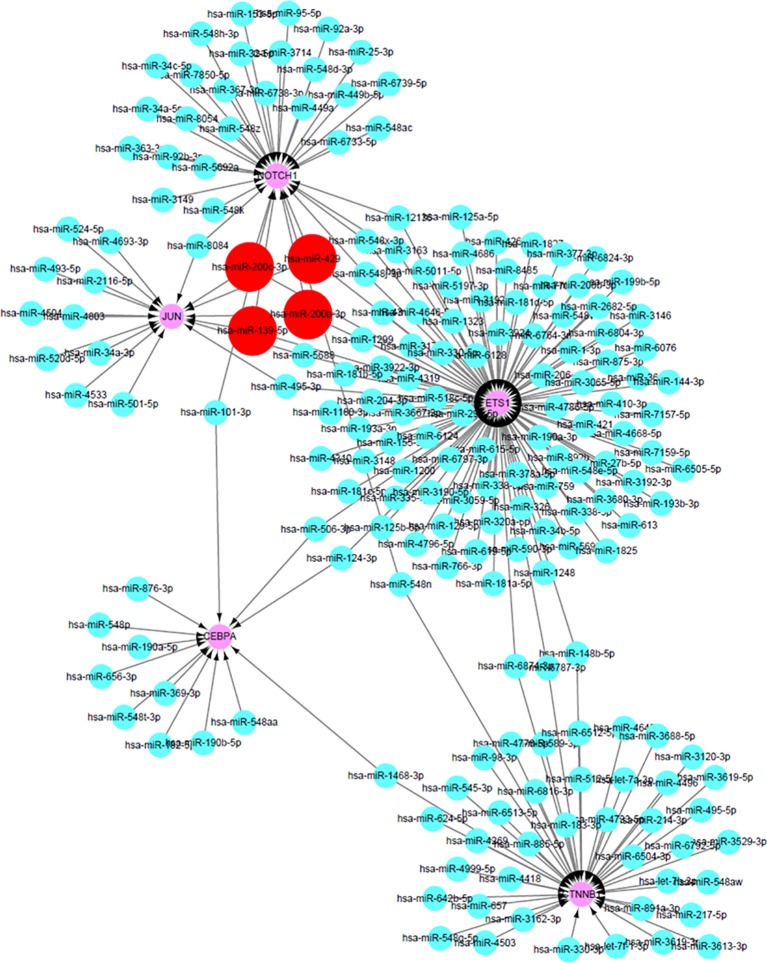
The construction of miRNA–mRNA network of hub genes in OSCC. The red circles predicted the potential miRNAs that can regulate multiple hub genes in OCSS.

As the DEGs in cisplatin-resistant OSCC cells were closely related to tumor-related miRNA, miRDB database was used to predict potential miRNAs that might participate in the transcriptional regulation of the hub genes in this process. The prediction scores were also collected from the miRDB database, and the miRNA–mRNA with high score meant close potential function of miRNA in regulation of the target mRNA. After setting cutoff > 80, Cytoscape software was used to construct the miRNA–mRNA network (**Figure 5**). Interestingly, hsa-miR-200c-3p, hsa-miR-200b-3p, hsa-miR-429, and hsa-miR-139-5p could simultaneously regulate multiple hub genes, which may be the key miRNAs involved in this process. Interestingly, hsa-miR-200c-3p, hsa-miR-200b-3p, and hsa-miR-429 belong to miR-200 family members, with similar functions; suppression of ZEB1/2, followed by inhibition of epithelial–mesenchymal transition (EMT).

### The Expression Pattern of Hub Genes in OSCC

**Figure 6:**
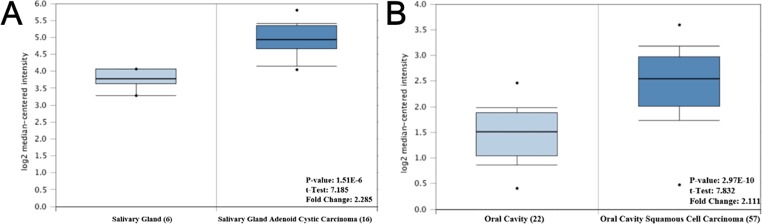
The mRNA expression pattern of hub genes in OSCC. **(A)** The expression of CTNNB1 was increased in OSCC tissues, compared with normal tissues. **(B)** The expression of ETS1 was increased in OSCC tissues, compared with normal tissues.

To investigate the potential function of the hub genes in OSCC, Oncomine database was used to analyze the difference in the expression levels of the hub genes. However, due to limited research on OSCC, only one study revealed that the expression of *CTNNB1* and *ETS1* in tumor tissues was higher than that in normal tissues, with 2.285 and 2.111 fold change, respectively, while no difference was found in the expression of *NOTCH1* and *JUN* genes in the two tissues (**Figure 6**).

### Survival Value of Hub Genes in OSCC

**Figure 7:**
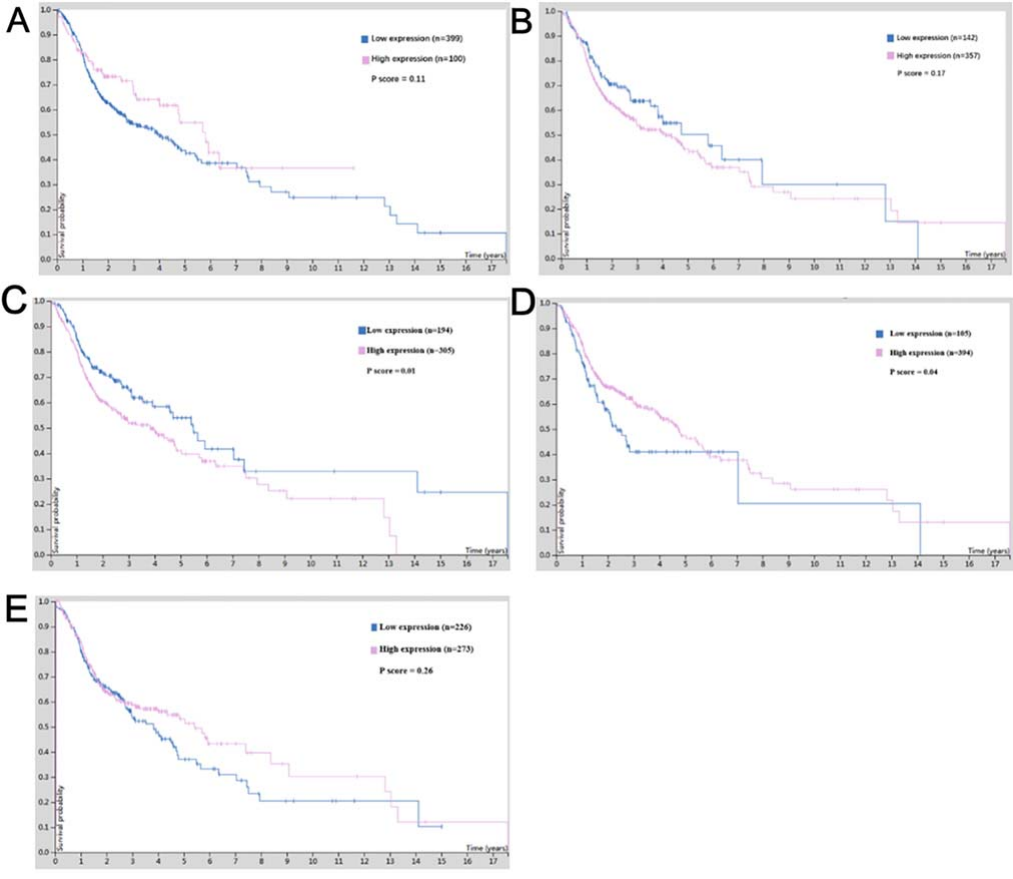
The survival value of the expression of hub genes in cisplatin-resistant OSCC. **(A)** NOTCH1. **(B)** JUN. **(C)** CTNNB1. **(D)** CEBPA. **(E)** ETS1.

For survival analysis, cBioportal based on TCGA database was used, which revealed that low expression of *CTNNB1* in patients with OSCC showed better overall survival (*p* = 0.01) (**Figure 7C**), and low expression of *CEBPA* predicted poor overall survival in OSCC patients (*p* = 0.04) (**Figure 7D**). Although the expression of other hub genes did not show a significant relationship with the survival status of OSCC patients (*p* > 0.05), the OSCC patients with high expression of *NOTCH1* (**Figure 7A**) and *ETS1* (**Figure 7E**) or low expression of *JUN* (**Figure 7B**) tended to have long lifespans.

**Figure 8:**
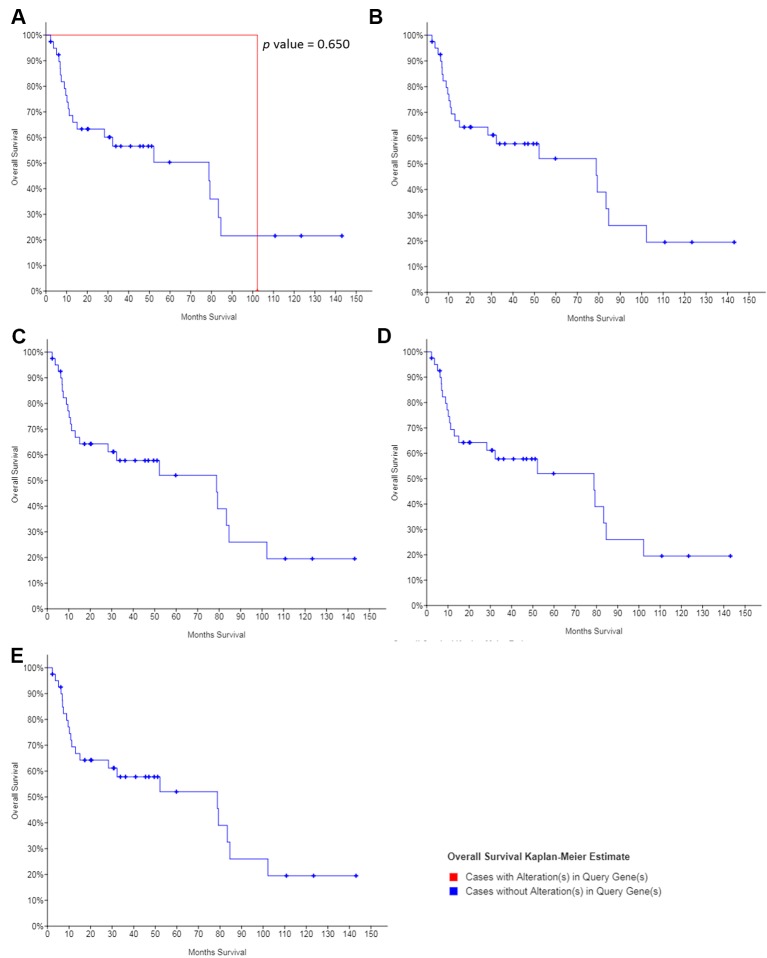
The survival value of the mutations of hub genes in cisplatin-resistant OSCC. **(A)** NOTCH1. **(B)** JUN. **(C)** CTNNB1. **(D)** CEBPA. **(E)** ETS1.

The dataset obtained from MD Anderson, Cancer Discov 2013, showed that the median overall survival of all OSCC patients was 78.8 months. Except for *NOTCH1*, no mutation was found in the other hub genes in the OSCC patients. And the mutations in *NOTCH1* showed no significant association with the overall survival of patients with OSCC (**Figure 8**), suggesting that the regulation, without mutation of the hub genes was the main mechanism of cisplatin resistance in OSCC.

## Discussion

Worldwide, OSCC is an important public health issue with limited therapy strategies and researches; systemic drug resistance has aggravated this situation. In this study, high-throughput screening was used to explore the potential genes involved in cisplatin resistance of OSCC, and *NOTCH1*, *JUN*, *CTNNB1*, *CEBPA*, and *ETS1* were identified as the hub genes in the occurrence of cisplatin resistance. These genes were found to be regulated by the members of the miR-200 family. Regulation of the corresponding hub genes by miRNAs may reverse cisplatin resistance of OSCC, and the sensitivity of tumor cells to cisplatin maybe restored; thus, providing a novel potential target for anticancer therapy.

Studies have shown that changes in NOTCH signaling pathway are associated with many human cancers (Villanueva et al., 2012). *NOTCH1* is reported to be both a tumor suppressor gene and a tumor oncogene. The tumorigenic or anti-tumor activity of NOTCH family members in different types of tumors displays its role in promoting or inhibiting the undifferentiated state of stem cells in the corresponding tissues (Wang et al., 2012). Carcinogenic action of NOTCH has been found in many cancers, including non-small cell lung cancer (Lenhart et al., 2015), acute T lymphoblastic leukemia (Weng et al., 2004), and malignant gliomas (Purow et al., 2005). In contrast, *NOTCH1* signaling is inhibited in neuroendocrine tumor cells, including small cell lung cancer (Platta et al., 2008). This suggests that induction of *NOTCH1* expression is an effective strategy for treating these tumors. NOTCH signaling pathway is also involved in chemotherapy resistance. For example, *NOTCH1* plays an important role in cisplatin resistance mechanism of head and neck squamous cell tumor, colorectal tumor, ovarian cancer (Wang et al., 2010), and other malignant tumors. In this study also, expression of *NOTCH1* gene was found to be significantly inhibited in cisplatin-resistant OSCC cell lines as compared to that in normal or tumor tissues, but no effect was observed on the overall survival of patients. These results suggest that NOTCH1 signaling molecules may be involved in different biological processes of malignant tumor development through different molecular pathways, and could play an important role in resistance of OSCC against cisplatin and other chemotherapy drugs.

*JUN* is a protein-coding gene and has no introns; it is located in 1p32-p31: a chromosomal region involved in human malignant translocations and deletions (Fazal et al., 2017) JUN-related diseases, include sarcomas and whooping cough (Syc-Mazurek et al., 2017). *JUN* is involved in the following pathways: apoptosis regulation, signal transduction, tacrolimus/cyclosporine pathway, and pharmacodynamics. *JUN* is also associated with sequence-specific DNA binding (GO annotation). In this study, although *JUN* molecular expression was significantly changed, its correlation with malignant tumor tissues and its influence on patient survival were not found. Therefore, its function and molecular mechanism will be explored in future studies.

The protein encoded by *CTNNB1* is a part of the protein complex that forms the adhesive junctions. Adhesion is necessary to create and maintain the epithelial layer (Li et al., 2017a). The coding proteins, which also include the actin cytoskeleton, are responsible for signaling contact inhibition, and once the upper cortex completes signaling, the cell stops dividing. Finally, the protein binds to the product of the *APC* gene, which is mutated in colorectal adenomatous polyposis. The mutation is a cause of colorectal cancer, hairy tumors, medulloblastoma, and ovarian cancer (Li et al., 2017b). Selective splicing of *CTNNB1* RNA leads to multiple transcript variants. Diseases associated with *CTNNB1*, include hairy tumors and intellectual disability, both being 19 autosomal dominant (Lee et al., 2018). The pathways associated with *CTNNB1* are beta-adrenergic signaling and blood-brain barrier pathways. Because it inhibits the expression of downstream signals, GO annotations associated with it, include DNA-binding transcription factor activity and binding. In this study, it was found that the expression of *CTNNB1* in tumor tissues was significantly higher than that in normal tissues, and the survival period of patients with high expression of *CTNNB1* was significantly shortened. These results suggest that *CTNNB1* also plays an important role in the occurrence and development of OSCC, but the mechanism of its influence on cisplatin chemotherapy resistance needs to be further studied and explored.

CEBPA is an intron-free transcription factor that contains a basic leucine zipper domain and recognizes the CCAAT motif in the target gene promoter (Mannelli et al., 2017). The coding proteins act in homodimers and heterodimers with CCAAT or enhancer binding proteins, beta and gamma. The activity of CEBPA protein regulates the expression of genes, which are involved in cell cycle regulation and weight balance. Mutation in the *CEBPA* gene has been linked to acute myeloid leukemia (Avellino et al., 2016). *CEBPA* mutations are particularly associated with cytogenetically-normal AML (Taskesen et al., 2011). *CEBPA* is necessary for granulocyte formation in mice. Mutations in *CEBPA* are associated with longer survival of OSCC patients. CEBPA-related diseases, include leukemia, acute myeloid leukemia, and myeloid leukemia. The pathways associated with *CEBPA* are adenoid cystic carcinoma and the adipogenesis pathway. CEBPA has important DNA binding transcription factor activity and can bind to sequence specific DNA. However, there are no relevant studies on *CEBPA* and cisplatin resistance of OSCC at present. In the current study, we found that high expression of *CEBPA* is closely related to the prognosis of OSCC patients.

*ETS1* is a member of the encoding transcription factor ETS family, which has a conserved DNA binding domain of ETS that recognizes the hub consistent DNA sequence GGAA/T in the target gene (Poon and Kim, 2017). These proteins act as transcriptional activators or inhibitors of many genes and are involved in stem cell development, cell aging and death, and tumorigenesis. Splicing transcriptional variants encoding different subtypes have also been previously described. Jacobsen syndrome and estrogen receptor negative breast cancer are the diseases associated with *ETS1* (Carpinelli et al., 2015). The pathways involving *ETS1* include focal adhesion and focal adhesion kinase mediated signal transduction events. The gene also has important DNA-binding transcription factor activity and transcription factor binding. We found that *ETS1* is an important cisplatin resistant gene based on high-throughput data analysis, PPI network, and expression verification. Studies have shown that overexpression of *ETS1* induces IKK alpha mRNA and protein expression as well as IKK alpha activity (Gu et al., 2004). In a previous study, ETS1 protein expression and IKK alpha were significantly upregulated in 231 cisplatin-resistant cell lines. Inhibition of *ETS1* expression has been reported to enhance cisplatin sensitivity of resistant cell lines. *ETS1* knockout increases the stability of cisplatin in mouse xenograft models (Zhang et al., 2018). These results are similar to the results obtained in the current study. *ETS1* was highly expressed in cisplatin-resistant OSCC cell lines as compared to that in the normal tissues; ETS1 was highly expressed in tumor tissues, suggesting that it is an important molecule in this process.

Based on previous studies on hub genes and members of the miR-200 family, miR-200b/a/429 transcription is known to be regulated by different transcriptional factors in tissue-specific manner (Kim et al., 2011b). ZEB1/2 is the classical target gene of miR-200s, and many other potential factors have also been reported as the genes regulated by miR-200s (Nagalla et al., 2011). In the current study, new potential target genes were reported as the hub genes in cisplatin-resistant OSCC cells. In 2018, Liu *et al*. reported a smart miRNA-reporter gene for *in vitro* and *in vivo* imaging of biogenesis of miRNA and their related functions (Liu et al., 2018). Further study involving the reporter system could be helpful in investigation of the relationship between miR-200s and the hub genes in OSCC. And as the researches related to OSCC are limited, the relationship between the expression of hub genes and clinicopathological parameters in OSCC patients will be collected and analyzed in the further, to confirm their roles in the occurrence of cisplatin resistance in OSCC.

## Conclusion

We found that *NOTCH1*, *JUN*, *CTNNB1*, *CEBPA*, and *ETS1* were the key genes regulating cisplatin resistance in OSCC drug-resistant cell lines, and the miR-200 family may be capable of reversing OSCC cell resistance by regulating *NOTCH1*, *JUN*, and *ETS1*, which could also act as potential targets for treating cisplatin resistant OSCC patients.

## Data Availability Statement

GSE111585 and GSE115119 were downloaded from Gene Expression Omnibus.

## Author Contributions

JL conceptualized and designed the study. W-TC, H-TW, and JL organized the database, searched literature, and structured and drafted the manuscript. G-WL, J-XS, Q-QY, M-LZ, and W-JC analyzed and interpreted the data, and drafted and revised the manuscript. JL revised the original manuscript. All authors contributed to manuscript revision and read and approved the submitted version.

## Funding

This work was supported by the National Natural Science Foundation of China (Nos. 81501539 and 81320108015), the Natural Science Foundation of Guangdong Province (No. 2016A030312008), and Li Ka Shing Foundation Grant for Joint Research Program between Shantou University and Technion-Israel Institute of Technology (No. 43209501).

## Conflict of Interest

The authors declare that the research was conducted in the absence of any commercial or financial relationships that could be construed as a potential conflict of interest.

## Supplementary Material

Supplemental Figure 1 The PPI network of DEGs in cisplatin-resistant DEGs. **(A)** The PPI network of up-regulated DEGs. **(B)** The PPI network of down-regulated DEGs.

## References

[B1] Avellino R. Havermans M. Erpelinck C. Sanders M. A. Hoogenboezem R. van de Werken H. J. (2016). An autonomous CEBPA enhancer specific for myeloid-lineage priming and neutrophilic differentiation. Blood 127 (24), 2991–3003. 10.1182/blood-2016-01-695759 26966090 PMC5043424

[B2] Carpinelli M. R. Kruse E. A. Arhatari B. D. Debrincat M. A. Ogier J. M. Bories J. C. (2015). Mice haploinsufficient for ETS1 and FLI1 display middle ear abnormalities and model aspects of Jacobsen syndrome. Am. J. Pathol. 185 (7), 1867–1876. 10.1016/j.ajpath.2015.03.026 26093983

[B3] Fazal S. V. Gomez-Sanchez J. A. Wagstaff L. J. Musner N. Otto G. Janz M. (2017). Graded elevation of c-Jun in Schwann cells *in vivo*: gene dosage determines effects on development, remyelination, tumorigenesis, and hypomyelination. J. Neurosci. 37 (50), 12297–12313. 10.1523/JNEUROSCI.0986-17.2017 29109239 PMC5729195

[B4] Gao J. Aksoy B. A. Dogrusoz U. Dresdner G. Gross B. Sumer S. O. (2013). Integrative analysis of complex cancer genomics and clinical profiles using the cBioPortal. Sci. Signal 6 (269), pl1. 10.1126/scisignal.2004088 23550210 PMC4160307

[B5] Gu L. Zhu N. Findley H. W. Woods W. G. Zhou M. (2004). Identification and characterization of the IKKalpha promoter: positive and negative regulation by ETS-1 and p53, respectively. J. Biol. Chem. 279 (50), 52141–52149. 10.1074/jbc.M407915200 15469934

[B6] Hong L. Yang Z. Ma J. Fan D. (2013). Function of miRNA in controlling drug resistance of human cancers. Curr. Drug Targets 14 (10), 1118–1127. 10.2174/13894501113149990183 23834156

[B7] Huang da W. Sherman B. T. Lempicki R. A. (2009). Systematic and integrative analysis of large gene lists using DAVID bioinformatics resources. Nat. Protoc. 4 (1), 44–57. 10.1038/nprot.2008.211 19131956

[B8] Kanehisa M. Furumichi M. Tanabe M. Sato Y. Morishima K. (2017). KEGG: new perspectives on genomes, pathways, diseases and drugs. Nucleic Acids Res. 45 (D1), D353–D361. 10.1093/nar/gkw1092 27899662 PMC5210567

[B9] Kim S. Y. Nam S. Y. Choi S. H. Cho K. J. Roh J. L. (2011a). Prognostic value of lymph node density in node-positive patients with oral squamous cell carcinoma. Ann. Surg. Oncol. 18 (8), 2310–2317. 10.1245/s10434-011-1614-6 21336511

[B10] Kim T. Veronese A. Pichiorri F. Lee T. J. Jeon Y. J. Volinia S. (2011b). p53 regulates epithelial–mesenchymal transition through microRNAs targeting ZEB1 and ZEB2. J. Exp. Med. 208 (5), 875–883. 10.1084/jem.20110235 21518799 PMC3092351

[B11] Lee Y. H. Huang W. C. Hsieh M. S. (2018). CTNNB1 mutations in basal cell adenoma of the salivary gland. J. Formos. Med. Assoc. 117 (10), 894–901. 10.1016/j.jfma.2017.11.011 29224720

[B12] Leemans C. R. Braakhuis B. J. Brakenhoff R. H. (2011). The molecular biology of head and neck cancer. Nat. Rev. Cancer 11 (1), 9–22. 10.1038/nrc2982 21160525

[B13] Lenhart R. Kirov S. Desilva H. Cao J. Lei M. Johnston K. (2015). Sensitivity of small cell lung cancer to BET inhibition is mediated by regulation of ASCL1 gene expression. Mol. Cancer Ther. 14 (10), 2167–2174. 10.1158/1535-7163.MCT-15-0037 26253517

[B14] Li N. Xu Y. F. Li G. Q. Yu T. T. Yao R. E. Wang X. M. (2017a). Exome sequencing identifies a *de novo* mutation of CTNNB1 gene in a patient mainly presented with retinal detachment, lens and vitreous opacities, microcephaly, and developmental delay. Medicine 96 (20), e6914. 10.1097/Md.0000000000006914 28514307 PMC5440144

[B15] Li Y. K. Zhang F. Q. Yang D. H. (2017b). Comprehensive assessment and meta-analysis of the association between CTNNB1 polymorphisms and cancer risk. Biosci. Rep. 37 (6), BSR20171121. 10.1042/Bsr20171121 28963373 PMC5700267

[B16] Liang H. Gong F. Zhang S. Zhang C. Y. Zen K. Chen X. (2014). The origin, function, and diagnostic potential of extracellular microRNAs in human body fluids. Wiley Interdiscip. Rev. RNA 5 (2), 285–300. 10.1002/wrna.1208 24259376

[B17] Lim L. P. Glasner M. E. Yekta S. Burge C. B. Bartel D. P. (2003). Vertebrate microRNA genes. Science 299 (5612), 1540. 10.1126/science.1080372 12624257

[B18] Lin Z. Sun L. Xie S. Zhang S. Fan S. Li Q. (2018). Chemotherapy-induced long non-coding RNA 1 promotes metastasis and chemo-resistance of TSCC *via* the Wnt/beta-catenin signaling pathway. Mol. Ther. 26 (6), 1494–1508. 10.1016/j.ymthe.2018.04.002 29699939 PMC5986977

[B20] Liu Y. Sun J. Zhao M. (2017). ONGene, a literature-based database for human oncogenes. J. Genet. Genomics 44 (2), 119–121. 10.1016/j.jgg.2016.12.004 28162959

[B19] Liu J. Shen J. X. He Zhang G. J. (2018). Bioluminescence imaging for monitoring miR-200c expression in breast cancer cells and its effects on epithelial–mesenchymal transition progress in living animals. Mol. Imaging Biol. 20 (5), 761–770. 10.1007/s11307-018-1180-4 29532351

[B21] Mannelli F. Ponziani V. Bencini S. Bonetti M. I. Benelli M. Cutini I. (2017). CEBPA-double-mutated acute myeloid leukemia displays a unique phenotypic profile: a reliable screening method and insight into biological features. Haematologica 102 (3), 529–540. 10.3324/haematol.2016.151910 28250006 PMC5394975

[B22] Nagalla S. Shaw C. Kong X. Kondkar A. A. Edelstein L. C. Ma L. (2011). Platelet microRNA–mRNA coexpression profiles correlate with platelet reactivity. Blood 117 (19), 5189–5197. 10.1182/blood-2010-09-299719 21415270 PMC3109541

[B23] Ong W. Zhao R. Lui B. Tan W. Ebrahimi A. Clark J. R. (2016). Prognostic significance of lymph node density in squamous cell carcinoma of the tongue. Head Neck 38 (1), E859–E866. 10.1002/hed.24113 25917601

[B24] Petersen P. E. (2003a). Global framework convention on tobacco control: the implications for oral health. Community Dent. Health 20 (3), 137–138. 10.1126/science.106.2757.419 12940302

[B25] Petersen P. E. (2003b). Tobacco and oral health—the role of the World Health Organization. Oral Health Prev. Dent. 1 (4), 309–315. 15643759

[B26] Petersen P. E. (2005). Strengthening the prevention of oral cancer: the WHO perspective. Community Dent. Epidemiol. 33 (6), 397–399. 10.1111/j.1600-0528.2005.00251.x 16262606

[B27] Platta C. S. Greenblatt D. Y. Kunnimalaiyaan M. Chen H. (2008). Valproic acid induces Notch1 signaling in small cell lung cancer cells. J. Surg. Res. 148 (1), 31–37. 10.1016/j.jss.2008.03.008 18570928 PMC2900385

[B28] Poon G. M. K. Kim H. M. (2017). Signatures of DNA target selectivity by ETS transcription factors. Transcription 8 (3), 193–203. 10.1080/21541264.2017.1302901 28301293 PMC5501379

[B29] Purow B. W. Haque R. M. Noel M. W. Su Q. Burdick M. J. Lee J. (2005). Expression of Notch-1 and its ligands, Delta-like-1 and Jagged-1, is critical for glioma cell survival and proliferation. Cancer Res. 65 (6), 2353–2363. 10.1158/0008-5472.Can-04-1890 15781650

[B30] Rhodes D. R. Yu J. Shanker K. Deshpande N. Varambally R. Ghosh D. (2004). ONCOMINE: a cancer microarray database and integrated data-mining platform. Neoplasia 6 (1), 1–6. 10.1016/s1476-5586(04)80047-2 15068665 PMC1635162

[B31] Sakai N. S. Samia-Aly E. Barbera M. Fitzgerald R. C. (2013). A review of the current understanding and clinical utility of miRNAs in esophageal cancer. Semin. Cancer Biol. 23 (6 Pt B), 512–521. 10.1016/j.semcancer.2013.08.005 24013023

[B32] Syc-Mazurek S. B. Fernandes K. A. Libby R. T. (2017). JUN is important for ocular hypertension-induced retinal ganglion cell degeneration. Cell Death Dis. 8, e2945. 10.1038/Cddis.2017.338 28726785 PMC5550879

[B33] Szklarczyk D. Gable A. L. Lyon D. Junge A. Wyder S. Huerta-Cepas J. (2019). STRING v11: protein–protein association networks with increased coverage, supporting functional discovery in genome-wide experimental datasets. Nucleic Acids Res. 47 (D1), D607–D613. 10.1093/nar/gky1131 30476243 PMC6323986

[B34] Taskesen E. Bullinger L. Corbacioglu A. Sanders M. A. Erpelinck C. A. J. Wouters B. J. (2011). Prognostic impact, concurrent genetic mutations, and gene expression features of AML with CEBPA mutations in a cohort of 1182 cytogenetically normal AML patients: further evidence for CEBPA double mutant AML as a distinctive disease entity. Blood 117 (8), 2469–2475. 10.1182/blood-2010-09-307280 21177436

[B35] Thomas P. D. (2017). The gene ontology and the meaning of biological function. Methods Mol. Biol. 1446, 15–24. 10.1007/978-1-4939-3743-1_2 27812932 PMC6438694

[B45] Uhlen M. Zhang C. Lee S. Sjöstedt E. Fagerberg L. Bidkhori G. (2017). A pathology atlas of the human cancer transcriptome. Science 357 (6352). pii: eaan2507. 10.1126/science.aan2507 28818916

[B36] Villanueva A. Alsinet C. Yanger K. Hoshida Y. Zong Y. W. Toffanin S. (2012). Notch signaling is activated in human hepatocellular carcinoma and induces tumor formation in mice. Gastroenterology 143 (6), 1660–166+. 10.1053/j.gastro.2012.09.002 22974708 PMC3505826

[B38] Wang Z. Li Y. Ahmad A. Azmi A. S. Banerjee S. Kong D. (2010). Targeting Notch signaling pathway to overcome drug resistance for cancer therapy. Biochim. Biophys. Acta 1806 (2), 258–267. 10.1016/j.bbcan.2010.06.001 20600632 PMC2955995

[B37] Wang J. Sullenger B. A. Rich J. N. (2012). Notch signaling in cancer stem cells. Adv. Exp. Med. Biol. 727, 174–185. 10.1007/978-1-4614-0899-4_13 22399347

[B39] Weng A. P. Ferrando A. A. Lee W. Morris J. P. T. Silverman L. B. Sanchez-Irizarry C. (2004). Activating mutations of NOTCH1 in human T cell acute lymphoblastic leukemia. Science 306 (5694), 269–271. 10.1126/science.1102160 15472075

[B40] Wong N. Wang X. (2015). miRDB: an online resource for microRNA target prediction and functional annotations. Nucleic Acids Res. 43, D146–D152. 10.1093/nar/gku1104 25378301 PMC4383922

[B41] World Health O. (2003). World Health Assembly adopts historic tobacco control pact. Indian J. Med. Sci. 57 (8), 377–378. 10.1016/j.medmal.2005.06.009 14526788

[B42] Wu Q. Yang Z. Nie Y. Shi Y. Fan D. (2014). Multi-drug resistance in cancer chemotherapeutics: mechanisms and lab approaches. Cancer Lett. 347 (2), 159–166. 10.1016/j.canlet.2014.03.013 24657660

[B43] Zhang Y. Z. Wu J. J. Ye M. N. Wang B. Sheng J. Y. Shi B. L. (2018). ETS1 is associated with cisplatin resistance through IKK alpha/NF-kappa B pathway in cell line MDA-MB-231. Cancer Cell Int. 18, 86. 10.1186/s12935-018-0581-4 29950928 PMC6009945

[B44] Zhao M. Kim P. Mitra R. Zhao J. Zhao Z. (2016). TSGene 2.0: a literature-based database of tumor suppressor genes for pan-cancer analysis. Nucleic Acids Res. 44 (D1), D1023–D1031. 10.1093/nar/gkv1268 26590405 PMC4702895

