# Supplementary figures and images for: Analysis of the Differentially Expressed Genes Induced by Cisplatin Resistance in Oral Squamous Cell Carcinomas and Their Interaction
Source: Front Genet. 2020 Jan 23;10:1328. doi: 10.3389/fgene.2019.01328 (PMC6989555; doi:10.3389/fgene.2019.01328)

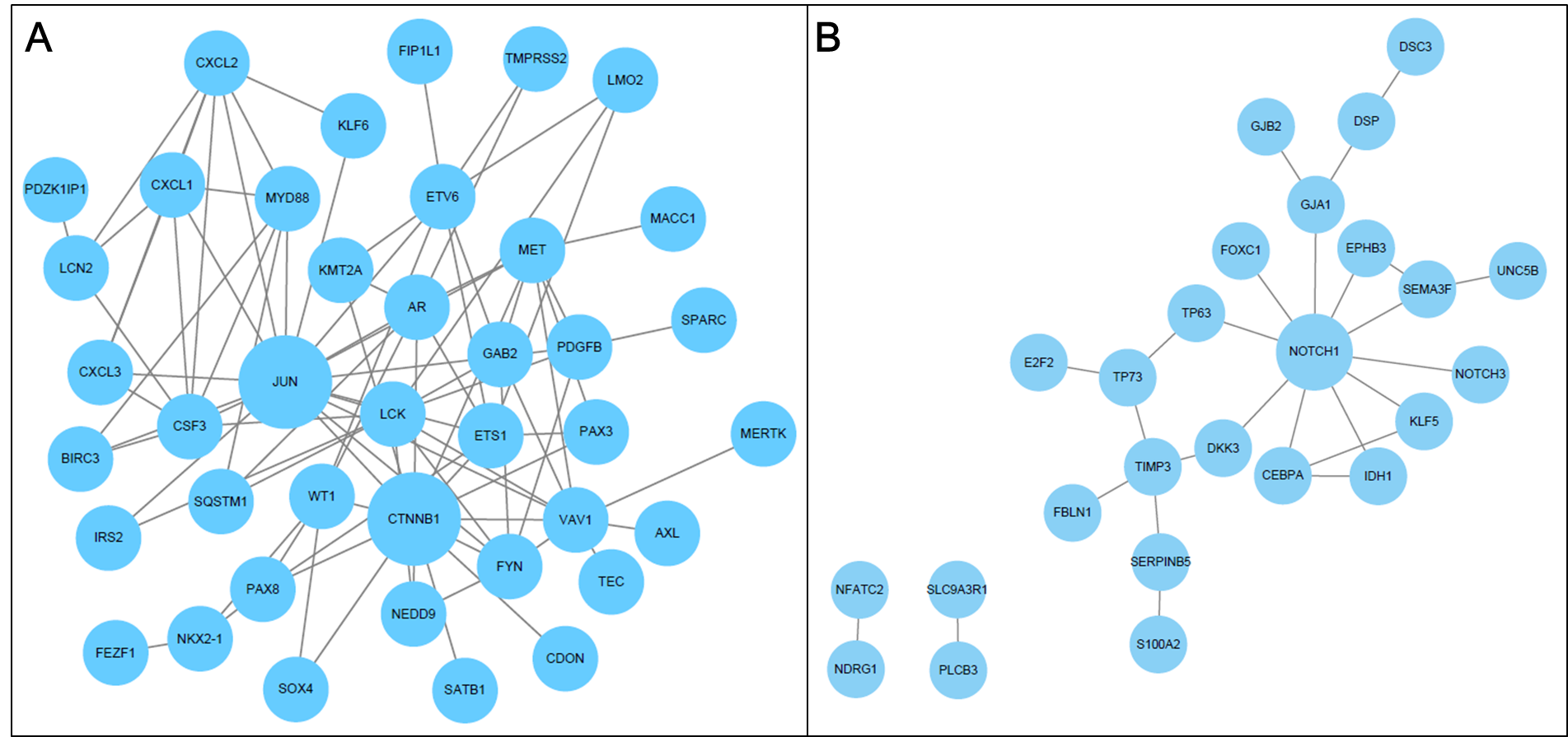

Supplement: Supplemental Figure 1 — The PPI network of DEGs in cisplatin-resistant DEGs. (A) The PPI network of up-regulated DEGs. (B) The PPI network of down-regulated DEGs. [file Image_1.tif]
